# COVID-19 and pulmonary embolism: Do not forget the association!

**DOI:** 10.1590/0037-8682-0234-2020

**Published:** 2020-06-08

**Authors:** Rachel Zerbini Mariano, Marcelo de Carvalho Ramos, Fabiano Reis

**Affiliations:** 1 Universidade Estadual de Campinas, Faculdade de Ciências Médicas, Departamento de Radiologia, Campinas, SP, Brasil.; 2 Universidade Estadual de Campinas, Faculdade de Ciências Médicas, Departamento de Medicina Interna, Campinas, SP, Brasil.

**FIGURE 1:**
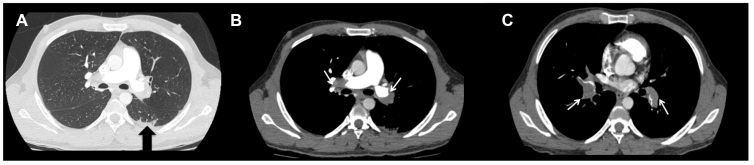
**(A)** Chest computed tomography, pulmonary window, axial, showing small areas of consolidation in the peripheral upper segment of the left lower lobe (black arrow), a typical finding in patients with COVID-19 pneumonia; **(B and C)** axial chest computed tomography with contrast in the mediastinal window. Large filling defects adherent to the walls of both the left and right main pulmonary arteries before their bifurcation (white arrows), an appearance compatible with pulmonary thromboembolism.

A 32-year-old man was admitted to the emergency department with a history of headache, fever, chills, dry cough, and fatigue. Chest computed tomography (CT) revealed predominantly peripheral consolidations involving all pulmonary lobes. These opacities were more exuberant in the peripheral upper segment of the left lower lobe (Figure 1 A, black arrow). Real-time polymerase chain reaction confirmed SARS-CoV-2 infection. An increase in blood D-dimer levels raised the suspicion of pulmonary thromboembolism, which was confirmed by CT pulmonary angiography (Figure 1B and C, white arrows).

In December 2019, a novel viral pneumonia (subsequently named coronavirus disease [COVID-19] pneumonia) emerged in Wuhan, China^1^ ^,^ ^2^. The main CT findings associated with COVID-19 pneumonia are bilateral, subpleural, ground-glass opacities with ill-defined margins, and a slight predominance in the right lower lobe, which includes predominantly peripheral ground-glass opacities, a crazy-paving pattern, and/or consolidation with air bronchograms of the middle and lower lung regions, usually with bilateral and multilobar involvement^1^ ^,^ ^2^. Abnormal CT pulmonary findings can be detected in asymptomatic patients and lung lesions can appear within 1-3 weeks of the onset of symptoms, peaking at around two weeks after onset^1^ ^,^ ^2^. Patients with diagnosed COVID-19 may also have acute pulmonary embolism^3^. In COVID-19 patients with raised D-dimer levels on admission or sudden clinical worsening, CT pulmonary angiography should be conducted. Although the etiology of pulmonary embolism associated with COVID-19 is still unclear^3^, adequate and accurate diagnosis can guide the appropriate treatment. 
